# Trends in Lipid‐Lowering Agent Consumption in Croatia: A 25‐Year Observational Study

**DOI:** 10.1002/prp2.70122

**Published:** 2025-05-19

**Authors:** Andrej Belančić, Marta Kučan Štiglić, Luka Prgomet, Ivan Pećin, Željko Reiner, Dinko Vitezić

**Affiliations:** ^1^ Department of Basic and Clinical Pharmacology and Toxicology University of Rijeka, Faculty of Medicine Rijeka Croatia; ^2^ Department of Internal Medicine, Division of Metabolic Diseases University Hospital Center Zagreb Zagreb Croatia; ^3^ Department of Internal Medicine, School of Medicine University of Zagreb Zagreb Croatia

**Keywords:** cholesterol, dyslipidemia, lipid‐lowering agents, statins

## Abstract

Cardiovascular diseases are the leading cause of mortality worldwide, with dyslipidemia as a major modifiable risk factor. This study aimed to assess 25‐year trends in lipid‐lowering agent consumption in Croatia from 2000 to 2023. We conducted a population‐based analysis using IMS and IQVIA databases, calculating drug utilization in defined daily doses per 1000 inhabitants per day (DDD/1000) and evaluating financial expenditures and prescribing patterns. Over the study period, total lipid‐lowering drug consumption increased more than 30‐fold, from 4.91 DDD/1000 in 2000 to 152.56 DDD/1000 in 2023. Statins, particularly atorvastatin and rosuvastatin, drove this trend, while the uptake of PCSK9 inhibitors and ezetimibe reflected an evolving therapeutic landscape. Financial expenditures peaked in 2010, declined until 2015, and rose again by 2023, with average drug prices per DDD decreasing significantly. The observed increase in lipid‐lowering therapy correlated with enhanced adherence to international guidelines and expanded patient access. However, administrative barriers and restrictive reimbursement policies continue to limit optimal utilization of newer agents. These findings underscore the importance of evidence‐based policy development to address clinical inertia and improve cardiovascular outcomes in Croatia.

## Graphical abstract

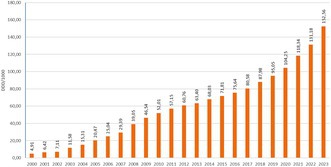

## 1 Introduction

Cardiovascular diseases (CVDs) remain the primary cause of mortality worldwide, with dyslipidemia being a well‐established modifiable risk factor [1]. Lipid‐lowering medicines (LLMs), particularly statins, are the cornerstone of dyslipidemia management, significantly reducing low‐density lipoprotein cholesterol (LDL‐C) levels and cardiovascular events. Over the past two decades, lipid‐lowering therapy has evolved due to new pharmacological developments, updated clinical guidelines, and changing prescription patterns [2, 3, 4].

Despite extensive global research on LLM utilization, long‐term population‐based analyses in Central and Eastern Europe, including Croatia, are scarce [5]. This study systematically evaluates the 25‐year trends in LLM consumption in Croatia, analyzing utilization in defined daily doses per 1000 inhabitants per day (DDD/1000), financial expenditures, and prescribing patterns. Additionally, we discuss and speculate on key determinants influencing these trends and assess their alignment with international guidelines and public health strategies.

Given the substantial increase in LLM use, it is crucial to explore the factors contributing to these trends [6]. Regulatory policies, pricing strategies, and guideline updates have all influenced prescribing behaviors, while growing awareness of cardiovascular risk has led to increased patient demand. The expanding role of combination therapies and novel LLMs, such as PCSK9 inhibitors and inclisiran, also reflects an evolving therapeutic landscape [7]. By assessing these patterns, this study provides a foundation for evaluating the impact of pharmacological interventions on cardiovascular health outcomes in Croatia.

The primary objective of this study is to assess the trends in LLM consumption in Croatia from 2000 to 2023, focusing on changes in prescribing patterns, financial expenditure, and key factors influencing these trends. The findings aim to provide insights into national prescribing practices and inform future healthcare policies on dyslipidemia management.

## 2 Materials and Methods

Data on the consumption of LLMs has been obtained from the databases widely used for pharmaceutical market analysis: IMS (International Medical Statistics) and IQVIA for Croatia for the period 2000–2023. According to the World Health Organization Collaborating Centre for Drugs Statistics Methodology, annual volumes of drugs are presented in defined daily doses/1000 inhabitants/day (DDD/1000). The ATC/DDD system is a tool used for drug utilization research to improve the quality of drug use and could provide a rough estimate of the proportion of the population within a defined area treated daily with certain drugs. For some of the data included in the analysis, the DDD values have changed over the years. For these drugs, the last approved DDD value was used for all years of the calculation.

According to the Guidelines for ATC classification and DDD assignment 2024 [8], the DDDs assigned for combination drugs are based on the main principle of counting the combination as one daily dose, regardless of the number of active ingredients included in the combination.

According to the Croatian Bureau of Statistics and its publications Census of Population, Households and Dwellings in the Republic of Croatia, the total population of Croatia in the years 2001, 2011, and 2021 is included in the calculation of the DDD/1000 value. For other years, it includes estimations about the total population calculated by the Croatian Bureau of Statistics and published on their web page. The population of Croatia, according to the 2001 census, decreased from 4 437 460 inhabitants to 3 871 833 in 2021, representing a decline of 12.75% [9].

Financial expenditure data are presented in Euro (€). The total financial consumption is presented in €, and the analyzed prices of each subgroup are presented in €/DDD. Changes in prescription patterns are measured with the index of change, a statistical measure of changes in a representative group of individual data points. It reflects changes in prescription in DDDs/1000 or in expenses in € in year 2023 compared with year 2000.

## 3 Results

**FIGURE 1:**
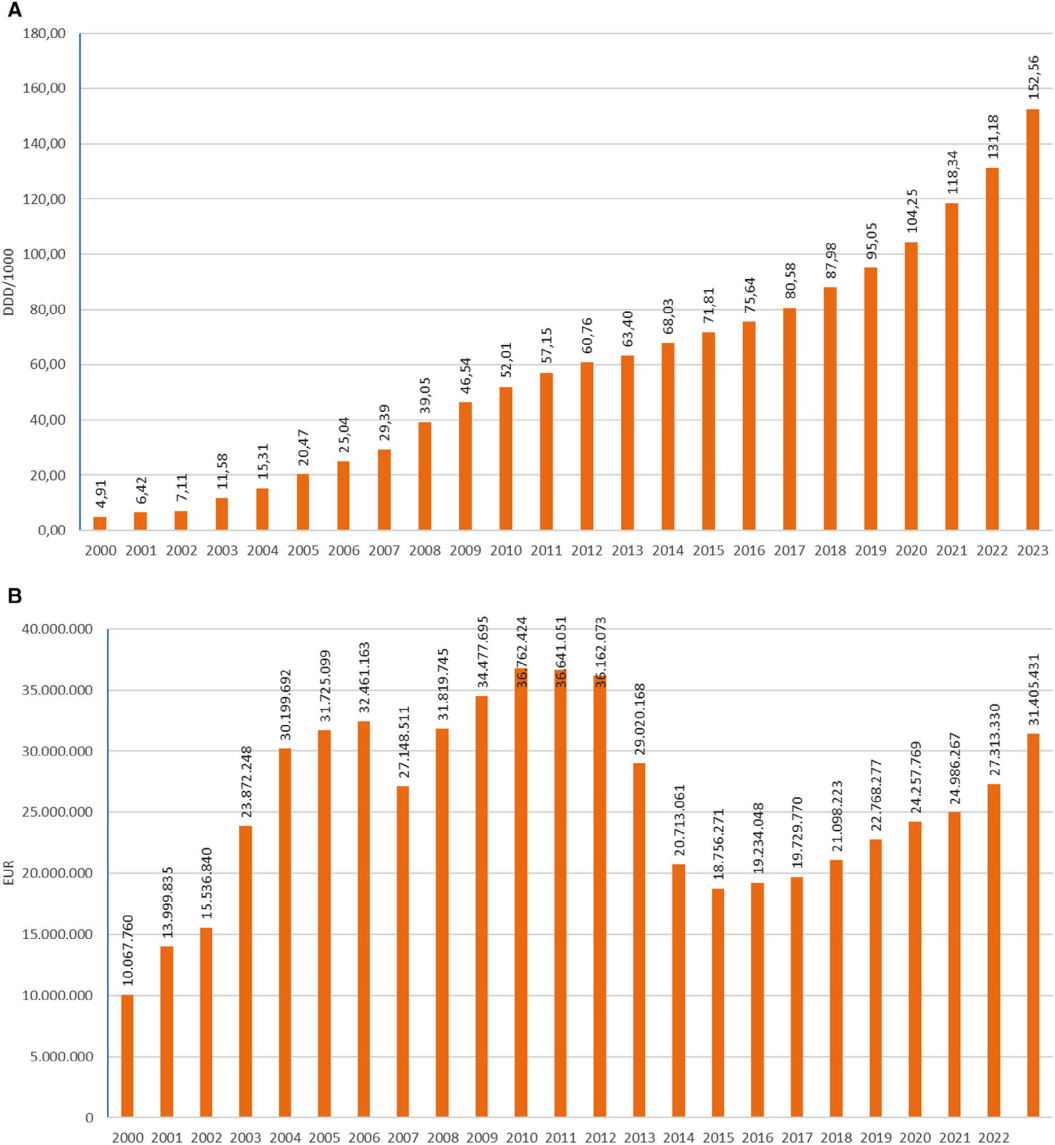
(A) Lipid‐lowering medicines consumption in Croatia during the period 2000–2023, in DDD/1000. (B) Lipid‐lowering medicines financial consumption in Croatia during the period 2000–2023, in EUR.

The total consumption of LLMs in Croatia was continuously rising from 4.91 DDD/1000 in 2000 to 152.56 DDD/1000 in 2023. Over the 24‐year period, the use of lipid‐lowering medicines, measured in DDD/1000, has increased more than 30 times. However, during the same timeframe, financial expenditure increased from 10.01 million euros in 2000 to a peak of 36.76 million euros in 2010, then fell to 18.76 million euros in 2015 and increased again to 31.41 million euros in 2023 (Figure 1B).

**FIGURE 2:**
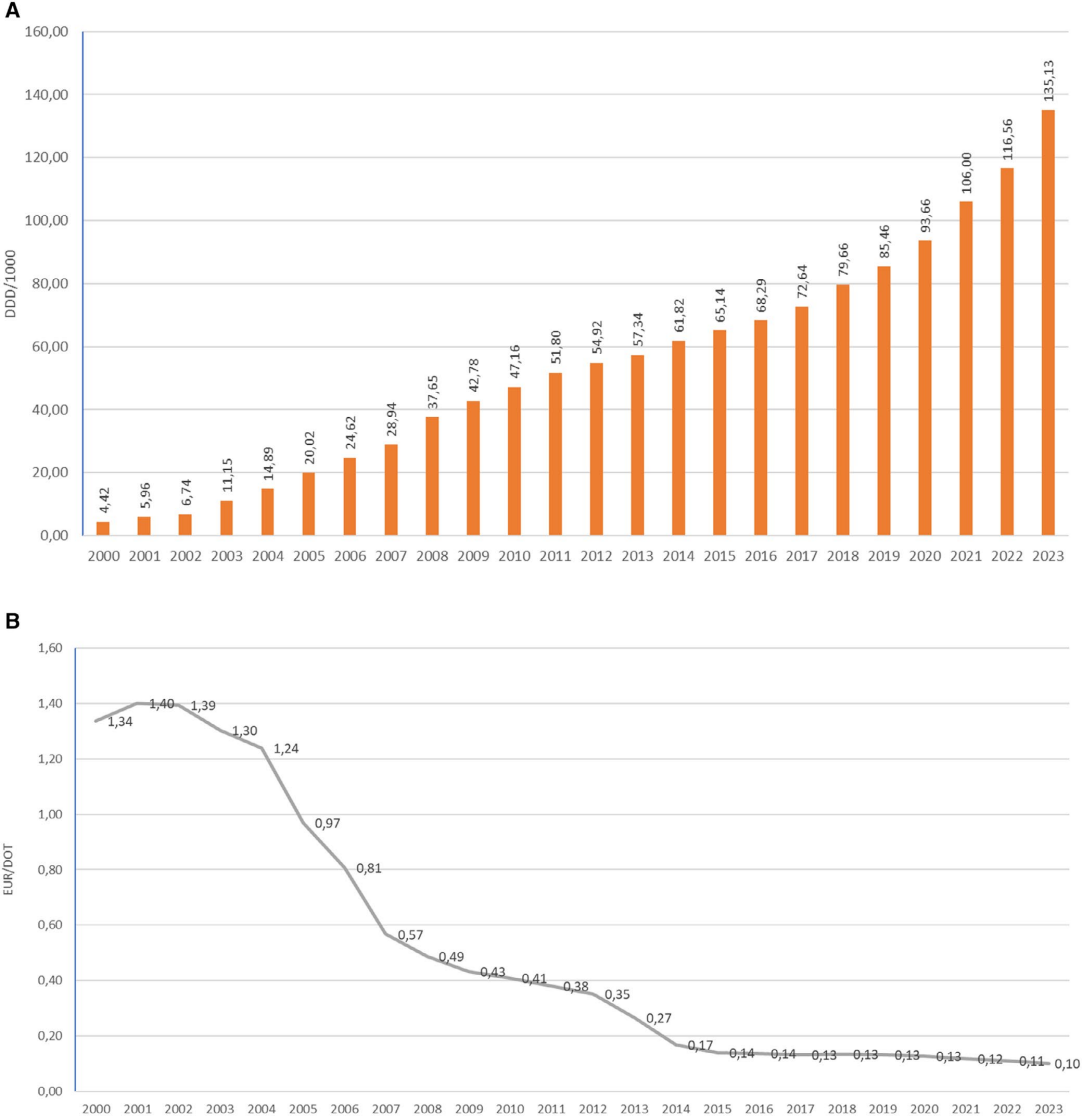
(A) HMG CoA reductase inhibitors consumption in Croatia during the period 2000–2023, in DDD/1000. (B) Prices per DDD for HMG CoA reductase inhibitors in Croatia during the period 2000–2023, in EUR.

The total consumption of HMG CoA reductase inhibitors in Croatia was continuously rising from 4.42 DDD/1000 in 2000 to 135.13 DDD/1000 in 2023. Over the 24‐year period, the usage of HMG CoA reductase inhibitors, measured in DDD/1000, has increased more than 30 times. The average price per DDD for HMG CoA reductase inhibitors was continuously decreasing from 1.54 EUR per DDD in 2000 to 0.10 EUR/DDD in 2023 (Figure 2A,B).

**FIGURE 3:**
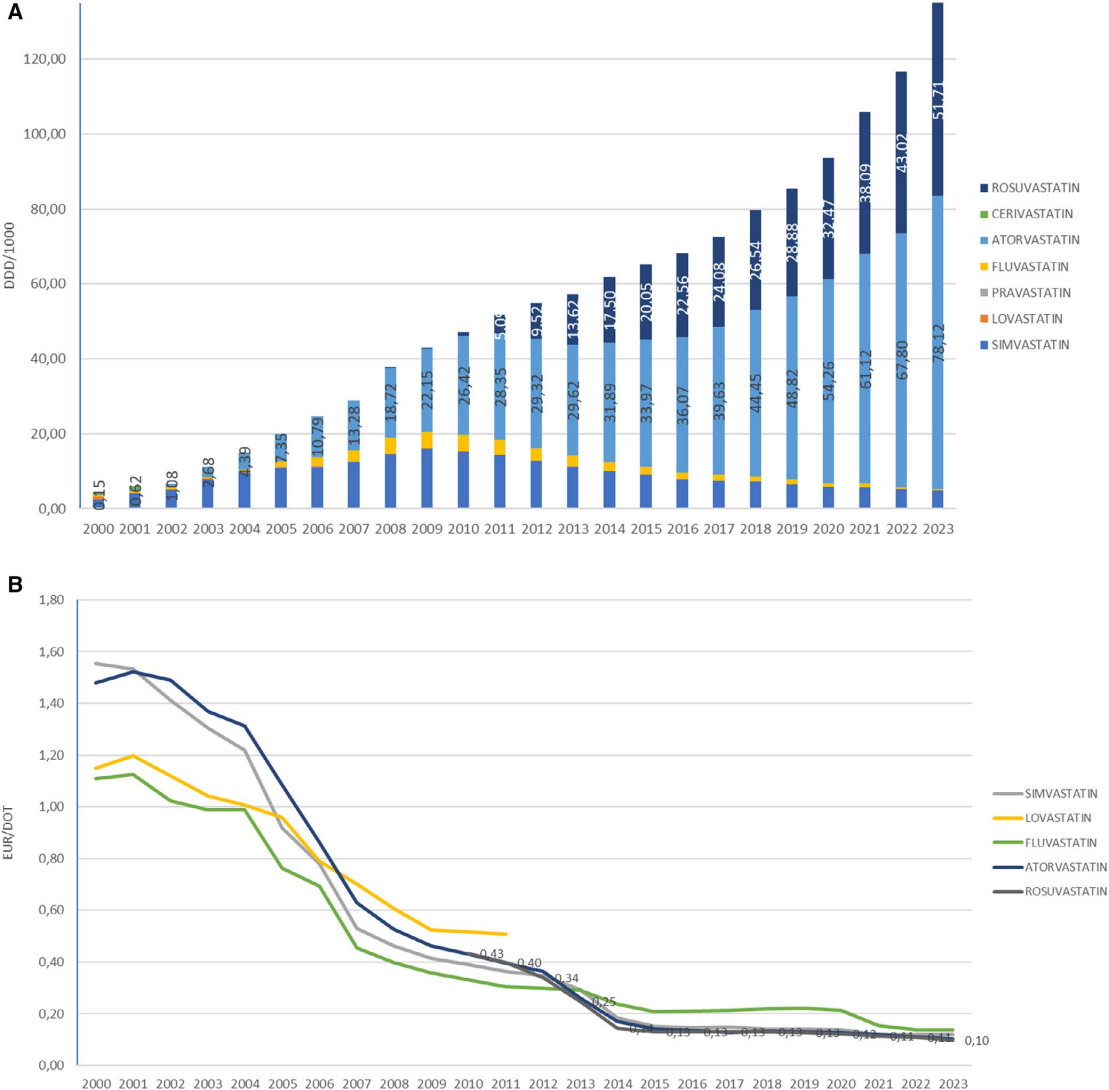
(A) Individual HMG CoA reductase inhibitors consumption in Croatia during the period 2000–2023, in DDD/1000. (B) Prices per DDD for individual HMG CoA reductase inhibitors in Croatia during the period 2000–2023, in EUR.

The most prescribed HMG CoA reductase inhibitor in Croatia from 2000 to 2005 was simvastatin (prescription raised from 2.50 to 10.90 DDD/1000 in 2005). In 2007, atorvastatin became the most prescribed HMG CoA reductase inhibitor (13.28 DDD/1000), compared with 12.40 DDD/1000 of simvastatin. Atorvastatin remained the drug with the highest prescription rate per DDD/1000 throughout the investigated period. The most significant growth was observed in the consumption of atorvastatin, which increased from 0.15 DDD/1000 in 2000 to 78.12 DDD/1000 in 2023. In contrast, the consumption of simvastatin decreased over the same period: its prescription decreased from 16.05 DDD/1000 in 2009 to 4.88 DDD/1000 in 2023. Rosuvastatin was introduced in 2003, but in 2010 it was approved by the FDA for the primary prevention of cardiovascular events, and from that time to 2023, it became the second most prescribed HMG CoA reductase inhibitor in Croatia, with a prescription of 51.71 DDD/1000 in 2023 (Figure 3).

The average price per DDD for most HMG CoA reductase inhibitors decreased over the period. The prices per DDD of the two most frequently prescribed drugs in a group, atorvastatin and rosuvastatin, were continuously decreasing. The price of atorvastatin was highest in 2001 (1.52 EUR/DDD) and was continuously dropping to 0.10 EUR/DDD in 2023, while the price of rosuvastatin dropped from 0.43 EUR per DDD in 2010 to 0.10 EUR/DDD in 2023 (Figure 3A,B).

**FIGURE 4:**
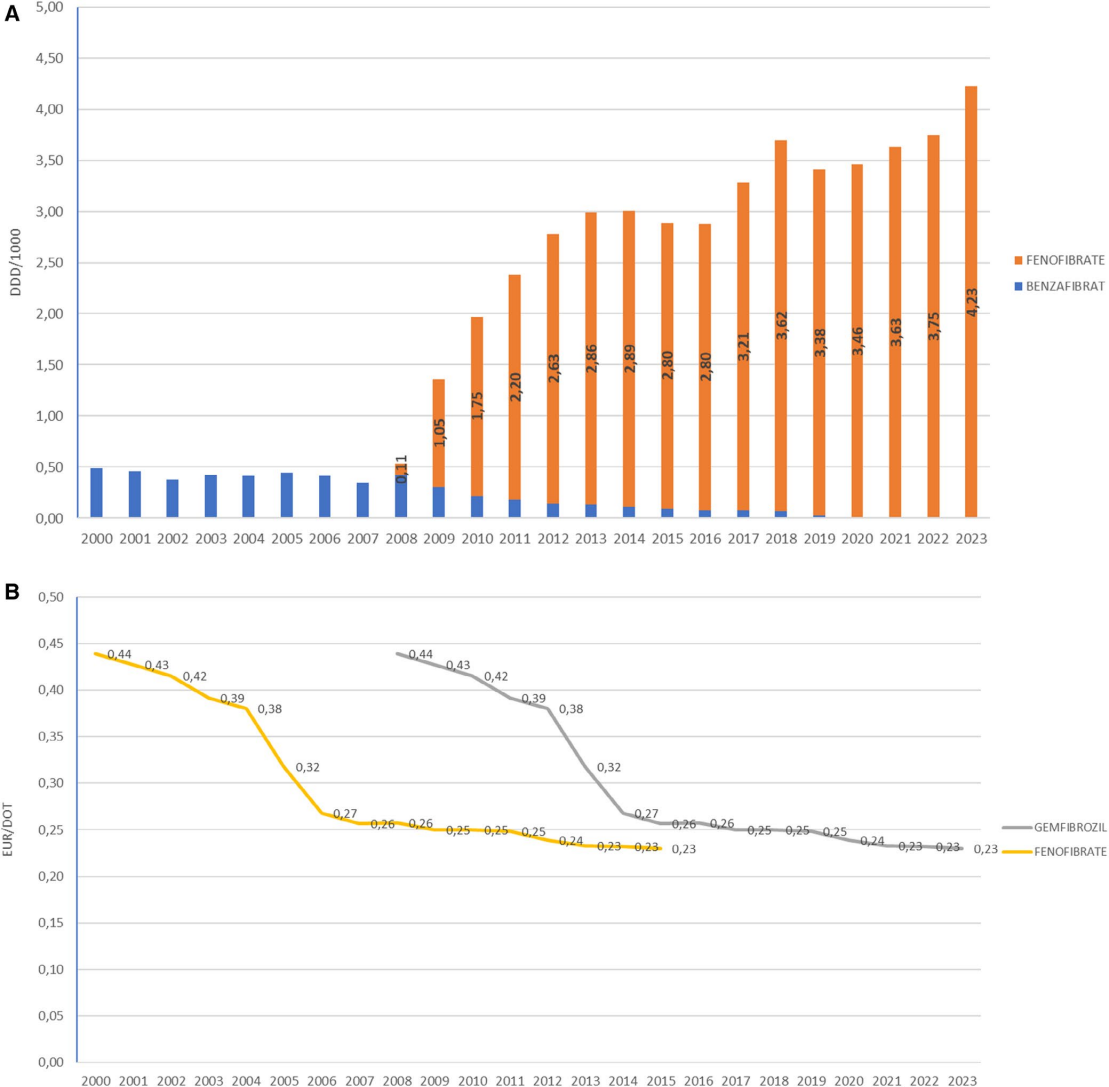
(A) Fibrates consumption in Croatia during the period 2000–2023, in DDD/1000. (B) Prices per DDD for fibrates in Croatia during the period 2000–2023, in EUR.

Among fibrates, the prescription of gemfibrozil decreased from 0.49 EUR/DDD in 2000 to 0.00 DDD/1000 in 2020. The prescription of fenofibrate increased from 0.11 DDD/1000 in 2008 to 4.23 DDD/1000 in 2023 (Figure 4A).

The average price per DDD for fenofibrate decreased from 0.44 EUR/DDD in 2008 to 0.23 EUR/DDD in 2023, while the average price for gemfibrozil decreased from 0.64 EUR/DDD in 2000 to 0.34 EUR/DDD in 2018 (Figure 4B).

**FIGURE 5:**
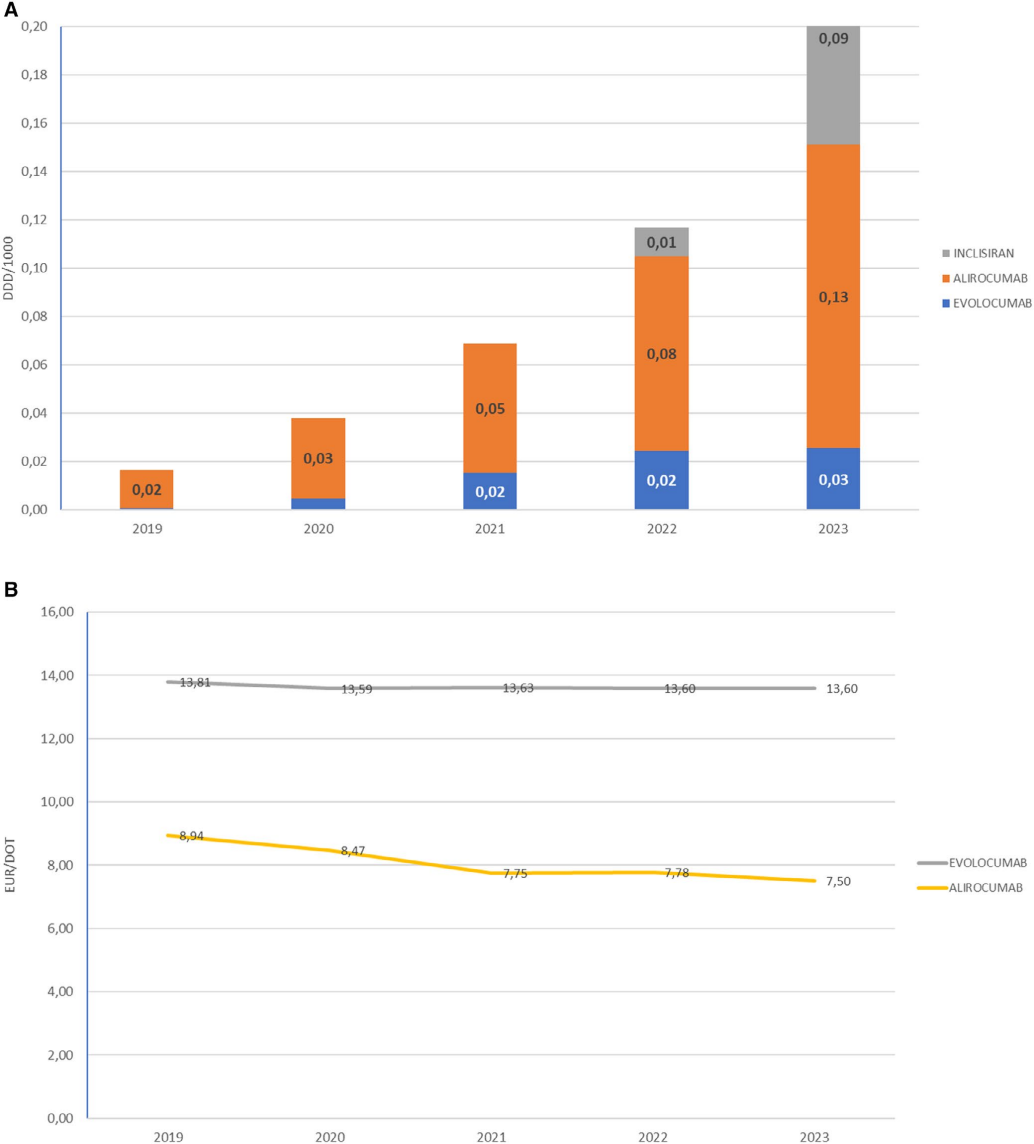
(A) PCSK9 inhibitors consumption in Croatia during the period 2000–2023, in DDD/1000. (B) Prices per DDD for PCSK9 inhibitors in Croatia during the period 2000–2023, in EUR.

PCSK9 inhibitors are classified among C10AX, Other lipid‐modifying agents. Alirocumab was approved by the FDA in 2015 but was introduced in Croatia in 2019, and in 2023, its prescription was 0.13 DDD/1000. The prescription of evolocumab was 0.03 in 2023, while the prescription of inclisiran, which received its first approval in December 2020 in the EU for use in adults with primary hypercholesterolaemia (heterozygous and non‐familial), started in Croatia in 2021. In 2023, its prescription was 0.09 EUR/DDD (Figure 5A). Ezetimibe was approved in the United States in 2002. The prescription of ezetimibe started in Croatia in 2007 (0.03 EUR/DDD), and it increased to 1.93 EUR/DDD in 2023.

The average price per DDD for evolucumab slightly decreased from 13.81 EUR/DDD in 2019 to 13.60 EUR/DDD in 2023; for alirocumab, it slightly decreased from 8.94 EUR/DDD in 2019 to 7.5 EUR/DDD in 2023, and for inclisiran, the price is 11.83 EUR/DDD in 2023 (Figure 5B). The price for ezetimibe decreased from 1.29 EUR/DDD in 2007 to 0.41 EUR/DDD in 2023.

**FIGURE 6:**
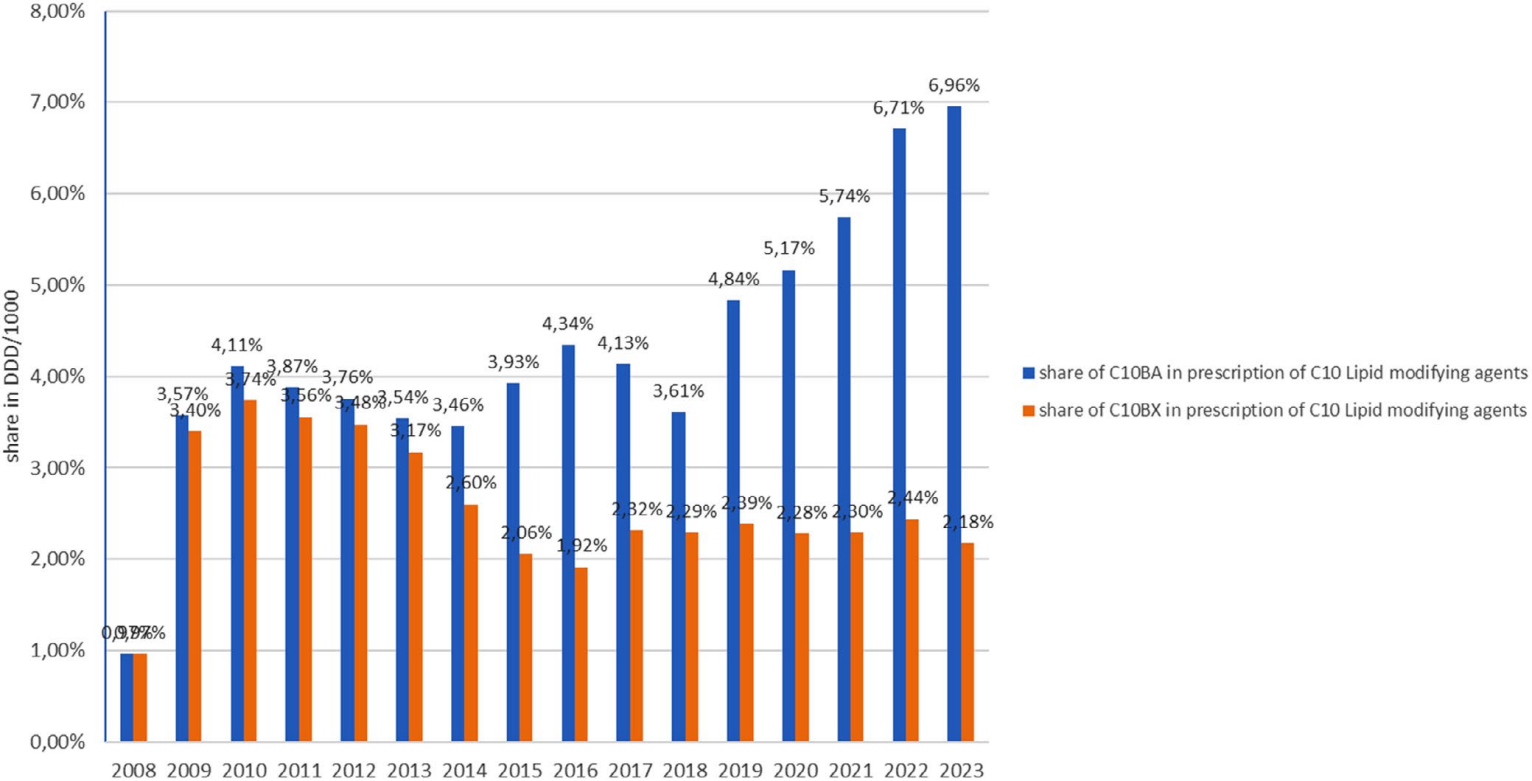
Share of prescribed lipid modifying drugs in combinations in Croatia from 2000 to 2023.

The subgroup C10B Lipid modifying agents in combinations consists of two subgroups: combinations of various lipid modifying agents (C10BA) and combinations of lipid modifying agents with other molecules (C10BX). Both were introduced to the Croatian market in 2008. The market share of combinations of various lipid modifying drugs (C10BA) in the total prescription of C10 Lipid modifying drugs was 6.96% in 2023, while the share of combinations of lipid modifying drugs with other molecules (C10BX) in the total prescription of C10 Lipid modifying agents was 3.32% in 2023 (Figure 6).

**FIGURE 7:**
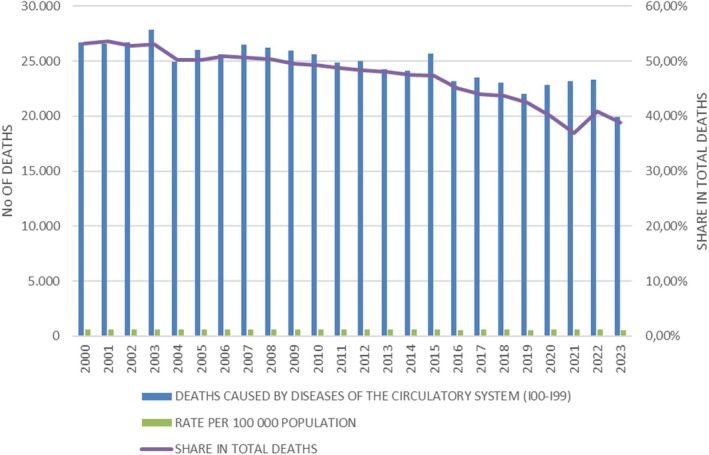
Deaths caused by diseases of the circulatory system in Croatia, from 2000 to 2023.

The Croatian Health Service Yearbook contains the most important data and indicators concerning the health condition indicators for the Croatian population. The total number of deaths caused by diseases of the circulatory system (ICD I00‐I99) decreased from 26.712 deaths in 2000 to 19.937 deaths in 2023 (Table 1 and Figure 7). The share of deaths caused by diseases of the circulatory system in total deaths was also constantly decreasing, from 53.16% in 2000 to 38.80% in 2023 [10].

**TABLE 1:** Deaths caused by diseases of the circulatory system in Croatia, from 2000 to 2023.

Year	Deaths caused by diseases of the circulatory system (I00–I99)	Total no of deaths	Rate per 100000 population	Share in total deaths
2000	26.712	50.246	609.67	53.16%
2001	26.542	49.552	598.13	53.56%
2002	26.698	50.569	601.65	52.80%
2003	27.872	52.575	628.11	53.01%
2004	24.959	49.756	562.46	50.16%
2005	26.029	51.790	586.57	50.26%
2006	25.611	50.378	577.15	50.84%
2007	26.506	52.367	597.32	50.62%
2008	26.235	52.151	591.22	50.31%
2009	25.976	52.414	586.48	49.56%
2010	25.631	52.096	580.18	49.20%
2011	24.841	51.019	564.21	48.69%
2012	24.988	51.710	585.53	48.32%
2013	24.232	50.386	569.40	48.09%
2014	24.112	50.839	568.90	47.43%
2015	25.694	54.205	611.24	47.40%
2016	23.190	51.542	555.54	44.99%
2017	23.504	53.477	569.86	43.95%
2018	23.048	52.706	563.82	43.73%
2019	22.020	51.794	541.66	42.51%
2020	22.817	57.023	563.71	40.01%
2021	23.184	62.712	597.68	36.97%
2022	23.303	56.979	578.45	40.90%
2023	19.937	51.275	516.50	38.80%

## 4 Discussion

To our knowledge, this is the first report on the use of LLMs in Croatia. Our dataset is unique as it includes all LLM prescriptions filled by pharmacies, including both state‐reimbursed and privately purchased medications.

From 2000 to 2023, we observed a consistent increase in the use of both statin and non‐statin LLMs. This trend highlights the enhanced implementation of international guideline recommendations and the growing demand for intensive lipid‐lowering therapy, driven by progressively lower LDL cholesterol (LDL‐C) targets for atherosclerotic cardiovascular disease (ASCVD) prevention.

Statins have been the first‐line treatment for dyslipidemia for over three decades. Over the years, Croatia has followed European trends in statin use. In the early 2000s, with the introduction of atorvastatin, its use significantly increased, while the use of low‐potency statins, such as simvastatin, decreased.

Between 2000 and 2023, atorvastatin consumption increased 520‐fold. A particularly noticeable increase was observed after 2019, when the latest ESC/EAS guidelines [11] further emphasized the “the lower, the better” approach, reducing the recommended LDL‐C target for high‐risk patients to below 1.4 mmol/L. This led to a substantial increase in prescriptions of all lipid‐lowering medications, including statins.

Rosuvastatin first became available in Croatia in 2008. Several studies, such as the PULSAR study [12], have shown that rosuvastatin and atorvastatin have comparable efficacy at equivalent doses in lowering LDL‐C. The most significant growth in rosuvastatin use in Croatia was observed in 2011, following the first ESC/EAS guidelines [13] that recognized it as equally potent as atorvastatin. Between 2010 and 2023, rosuvastatin consumption in Croatia increased 52‐fold. The price of statins in Croatia has continuously decreased. For example, the price of atorvastatin (EUR/DOT) decreased by 93% between 2000 and 2023.

Ezetimibe is the second‐line treatment for dyslipidemia, prescribed when statin therapy alone does not achieve target LDL‐C levels. The most influential study solidifying ezetimibe's role in dyslipidemia management was the IMPROVE‐IT study [14], published in 2015. Its findings were confirmed by the 2016 ESC/EAS guidelines [15]. Additionally, the increasing availability of fixed‐dose combinations of statins and ezetimibe has further contributed to its growing use.

Since 2015, Croatia has seen a consistent and significant rise in ezetimibe prescriptions, with consumption increasing 8‐fold (700%) over this period. The price of ezetimibe has followed a similar trend, with the most substantial price drop (68%) occurring between 2015 and 2023. A particularly notable increase in ezetimibe prescriptions has been observed since 2018, largely due to a Croatian Health Insurance Fund clause requiring the use of the maximum tolerated dose of statins in combination with ezetimibe as a prerequisite for introducing treatment with PCSK9 inhibitors. This policy passively increased ezetimibe prescriptions. It is worth noting that in June 2024, the Croatian Health Insurance Fund removed this ezetimibe clause, allowing general practitioners to prescribe ezetimibe without a specialist's recommendation. This restriction had been one of the main reasons for ezetimibe's initial underutilization.

PCSK9 inhibitors gained traction following the FOURIER [16] and ODYSSEY [17] trials, which demonstrated their efficacy in lowering LDL‐C and cardiovascular mortality. Although some meta‐analyses have questioned the incremental cardiovascular benefit of PCSK9 inhibitors when added to statin therapy [18], the 2019 ESC/EAS guidelines and prominent expert bodies, such as the International Lipid Expert Panel (ILEP), strongly recommend their use in certain high‐risk and all very high‐risk patients who fail to achieve LDL‐C targets despite maximally tolerated statin and ezetimibe therapy [11, 19].

Even though the impact on LDL‐C and the decrease in cardiovascular risk is evident, the cost‐effectiveness of PCSK9 inhibitors remains controversial, due to the prices of PCSK9 inhibitors being high since being introduced on the market [20]. PCSK9 inhibitor use in Croatia has aligned with guideline recommendations, with a notable increase in 2019, primarily driven by alirocumab, which saw a 6‐fold increase in consumption by 2023. In contrast, evolocumab has been prescribed significantly less, with dispensation rates four times lower than alirocumab in 2023.

Although alirocumab has been slightly cheaper than evolocumab in Croatia, its more frequent prescription is primarily due to its earlier market entry and stronger marketing campaign targeted at physicians, which contributed to its widespread use. One of the most significant barriers to the widespread prescription of PCSK9 inhibitors is the administrative burden, as each patient requires approval from the hospital drug committee. At this moment, despite indications supporting their use in all European and American guidelines, the Croatian Health Insurance Fund covers the cost of these drugs for only a small subset of patients, further limiting prescription rates. On a positive note, as observed with statins and ezetimibe, the cost of PCSK9 inhibitors is likely to decrease over time, and reimbursement policies may become more flexible, ultimately allowing physicians to prescribe them to a broader subset of patients.

After the first results on LDL‐C‐lowering effects from the ORION trials [21] were published in 2020, inclisiran entered the Croatian market in 2022. Within just 1 year, its consumption increased 9‐fold. It is currently prescribed to select patients at high and very high cardiovascular risk, most commonly those with familial hypercholesterolemia and additional risk factors or those who have recently experienced an acute coronary event. The full impact of inclisiran on cardiovascular outcomes in high‐risk patients remains to be seen, with the VICTORION‐2 PREVENT and ORION‐4 trials still ongoing. Their results will likely influence future inclisiran use [19, 22].

Fenofibrate is the only fibrate currently available in Croatia. While its use has steadily increased since its introduction to the market in 2008, it remains infrequently prescribed due to its primary effect on triglycerides rather than LDL‐C. This prescribing pattern aligns with current guidelines, which emphasize LDL‐C and apolipoprotein B‐containing lipoproteins as the main targets of lipid‐lowering therapy [11]. Moreover, current evidence indicates that fenofibrate, similar to other fibrates, does not significantly reduce cardiovascular risk and may be associated with an increase in overall mortality [23].

It is worth noting that bempedoic acid, a novel hypolipidemic agent, entered the Croatian market in 2025. Primarily used in statin‐intolerant patients, it is a prodrug activated in the liver, thereby avoiding muscle‐related side effects. The CLEAR OUTCOMES trial demonstrated its significant LDL‐C‐lowering efficacy in statin‐intolerant individuals [24], along with a reduction in the primary composite endpoint of major adverse cardiovascular events (MACE), including nonfatal myocardial infarction, nonfatal stroke, and coronary revascularization. Recent ILEP publications also highlight the potential of bempedoic acid to further reduce LDL‐C levels when added to statin and ezetimibe therapy in very‐high‐risk patients [19, 25]. Nonetheless, additional clinical trials are necessary to confirm its long‐term cardiovascular benefits, particularly as an add‐on therapy in high‐ and very‐high‐risk populations.

To the best of our knowledge, only a few similar population‐based analyses on LLM consumption exist, particularly in Europe. A study by Praskilevics et al. in 2023 [26] reported similar findings, primarily highlighting the increase in high‐potency statin and ezetimibe consumption over a 10‐year period from 2012 to 2021. Their study showed that statin consumption doubled, while ezetimibe use increased 25‐fold. However, the data in Praskilevics et al.'s study was based exclusively on state‐funded lipid‐lowering medications and used a different metric, defining consumption as the number of units dispensed. In contrast, our study used defined daily doses per 1000 inhabitants per day (DDD/1000), providing a different perspective on medication usage. A study by Kalinić et al. in 2023 [27] reported an almost threefold increase in lipid‐lowering medication use, with statins leading the way, showing a 163.07% increase in the analyzed period (2010–2019). Like our study, rosuvastatin had the highest expenditure rise, with its use increasing 1500‐fold. However, they did not analyze PCSK9 inhibitor use, likely because these drugs were not available in this period. García Rodríguez et al. in 2021 [28] analyzed the annual prevalence proportions of statin use in four well‐developed European countries (UK, Udine in Italy, the Region of Southern Denmark, and Spain) between 2010 and 2018. During this period, statin use remained stable in the UK, while it marginally increased in Spain, Udine, and the Region of Southern Denmark.

According to yet unpublished preliminary results of the EHUH‐2 study (Epidemiology of Hypertension in Croatia 2), clinical inertia among physicians regarding the prescription of lipid‐lowering medicines is alarmingly widespread in Croatia. Based on unofficial findings, the prevalence of dyslipidemia in Croatia exceeds 60% and only 4.2% of patients with dyslipidemia have adequately controlled LDL cholesterol levels.

In general, drug prescription patterns in Croatia are difficult to compare with those in other countries in the region and Europe overall, as they depend on national insurance policies and whether the state or other insuring companies and/or institutions reimburse medication costs. Prescription rates are also influenced by health literacy. Therefore, an initiative titled Hunt on the Silent Killer, with the missions 70/26 and Do You Know Your Number? is currently underway [29]. The aim is to educate both physicians and the general population about cardiovascular risk factors, which is expected to significantly improve adherence and reduce clinical inertia regarding lipid‐lowering therapy.

Lastly, the only available epidemiological data about the possible consequences of increased use of LLMs is from the Croatian Institute of Public Health, which collects data about leading causes of death (available through the publication Croatian Health Service Yearbook). There is a noticeable decrease or stagnation in the number of deaths caused by diseases of the circulatory system, which could be related to increased use of LLMs.

To deduce, the increase in consumption of LLMs can be divided into three main determinants: (i) increased prevalence; (ii) more access (more patients treated and therefore less undertreatment) and (iii) increased intensity of treatment on the same patient. But, as the source of the data for the study was total consumption, which is not linked to the individual patient, it is not possible to confirm that the increased prescription is correlated with more access.

### 4.1 Strengths and Limitations

The study is limited to a specific country (Croatia), but the analysis of the considerable amount of specific data we have performed allows to a certain extent the possibility for generalization and could be helpful for the international community. It is possible to identify the factors influencing prescription patterns (pricing policy changes could influence drug financial expenditure considerably, as well as therapeutic guidelines on dyslipidemia and pharmaceutical industry marketing activities) and implement them in the national healthcare system to predict or control changes in prescription patterns.

The source of the data were IMS and IQVIA databases that collect data from all existing wholesalers in Croatia, both for public and hospital pharmacies. However, the data on the consumption represent the total amount of drugs in pharmacies in Croatia during the investigated period. The data do not directly capture whether the medications are actually dispensed or prescribed to patients. This can lead to discrepancies between sales data and actual patient usage. These databases generally assume that the stock in pharmacies remains relatively constant, without taking into account the variations in inventory levels over time.

At the moment, this is the best possible source for drug use study in Croatia, because it is not possible to link specific drug prescriptions to specific patients. As the source of the data for the study was the data regarding total consumption and not individual patients, it is not possible to confirm that the increased prescription of C10 lipid‐modifying agents is correlated with more access (possible limitation of the study). The assumption is that access to medications is not a specific problem in Croatia because all citizens are insured by the National Health Insurance Fund and have full access to the drugs from the basic list.

## 5 Conclusions

Over the past 25 years, the consumption of LLMs in Croatia has increased more than 30‐fold, reflecting enhanced adherence to international guidelines and greater access to lipid‐modifying therapy. While the rise in statin use, particularly atorvastatin and rosuvastatin, has driven this trend, the adoption of newer agents such as PCSK9 inhibitors and ezetimibe indicates a shift towards more intensive lipid‐lowering strategies. The declining cost per defined DDD suggests that pricing policies and generic availability have played a pivotal role in shaping prescribing practices.

Despite these advancements, disparities in access to novel LLMs persist, partly due to restrictive reimbursement policies and administrative barriers. Future efforts should focus on optimizing treatment accessibility and adherence, addressing clinical inertia, and further integrating evidence‐based lipid management into routine clinical practice. The findings of this study contribute valuable insights into the long‐term evolution of lipid‐lowering therapy in Croatia and offer a foundation for policy development aimed at reducing the burden of cardiovascular disease.

## Author Contributions

Project administration: Andrej Belančić. Methodology: Marta Kučan Štiglić, Andrej Belančić, Dinko Vitezić. Investigation: all authors. Writing – original draft: Andrej Belančić, Marta Kučan Štiglić, Luka Prgomet. Writing – review and editing: all authors. Conceptualization; Andrej Belančić, Marta Kučan Štiglić, Dinko Vitezić. Supervision: Dinko Vitezić, Željko Reiner, Ivan Pećin. All authors have read and agreed to the published version of the manuscript.

## Ethics Statement

The authors have nothing to report.

## Conflicts of Interest

The authors declare no conflicts of interest.

## Data Availability

Available upon reasonable request sent to the corresponding author.
